# Forward-viewing echoendoscope-guided recanalization plus radial incision and cutting technique for rectal anastomotic atresia

**DOI:** 10.1055/a-2432-3391

**Published:** 2024-11-13

**Authors:** Xiao Li, Qingshan Pei, Shengqiang Zhao, Qian Ding, Zhen Li, Yongjun Shi

**Affiliations:** 1 34708 Department of Gastroenterology, Shandong Provincial Hospital Affiliated to Shandong First Medical University, Jinan, China

Rectal anastomotic atresia is rare in clinical practice and is challenging to manage using traditional approaches 1 2 3 4 . Here, we report successful recanalization utilizing a forward-viewing echoendoscope and endoscopic radial incision and cutting (ERIC) technique.

A 61-year-old man who had undergone laparoscopy-assisted radical resection combined with protective ileostomy and post-surgical chemoradiotherapy for rectal carcinoma was admitted to our hospital. Colonoscopy revealed a completely occluded anastomosis 10 cm from the anus, characterized by surgical staples and white scar ( Fig. 1 ).

**Fig. 1:**
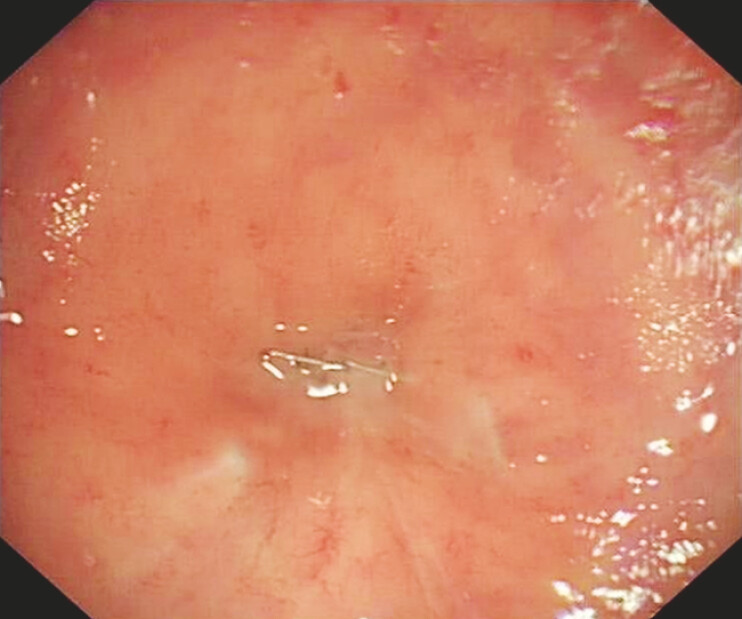
Complete obstruction of the anastomosis after surgery and chemoradiotherapy for rectal cancer.

To recanalize the lumen, the colonoscope was first inserted through a temporary stoma ( Video 1 ). A mixed solution of contrast medium and methylene blue was injected and revealed by X-ray fluoroscopy ( Fig. 2 ). Second, a forward-viewing echoendoscope (Olympus, Tokyo, Japan) was advanced to the obstruction site through the anus. The distal intestinal lumen was punctured with a 19-gauge needle (Cook Medical Inc., Bloomington, Indiana, USA) under the guidance of X-ray and endoscopic ultrasound (EUS) ( Fig. 3 ), which was confirmed by successfully withdrawing the mixed solution. Then, a 0.035-inch guidewire was inserted and retained, and the puncture needle was retrieved. Unfortunately, dilation using an 8.5-Fr bougie (Cook Medical Inc.) failed due to the staples and severe fibrosis. Alternatively, a 10-Fr cystotome (Cook Medical Inc.) was used to incise the occlusion, and then the ERIC technique was meticulously executed using a Multi-Function Knife (Anrui Medicine Co., Ltd., Hangzhou, China), allowing the passage of the colonoscope. The anastomotic stenosis was sequentially dilated to 15 mm with a balloon (Micro-Tech [Nanjing] Co., Ltd., Nanjing, China) ( Fig. 4 ). No severe immediate or delayed complications were observed during the procedure.

Forward-viewing echoendoscope-guided recanalization plus endoscopic radial incision and cutting technique in a patient with rectal anastomotic atresia.Video 1

**Fig. 2:**
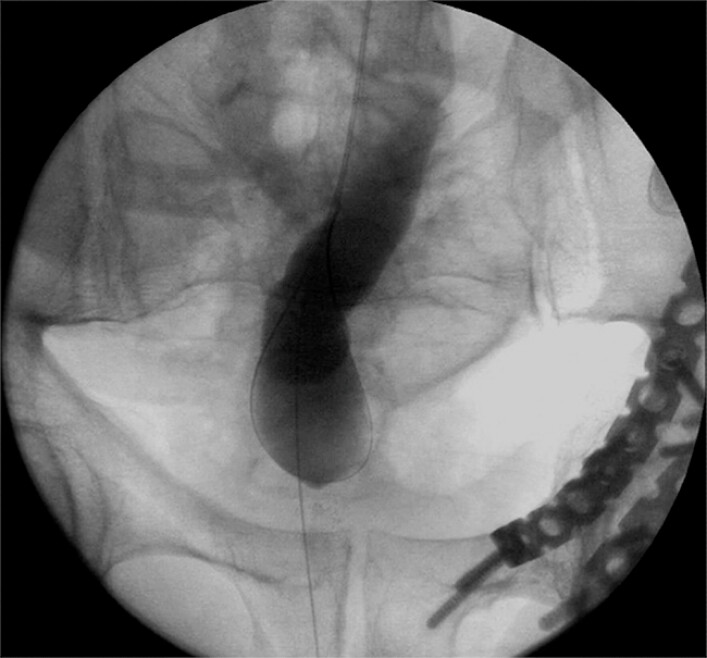
X-ray fluoroscopy showed the mixed solution of contrast medium and methylene blue in the intestinal cavity on the oral side of the obstruction.

**Fig. 3:**
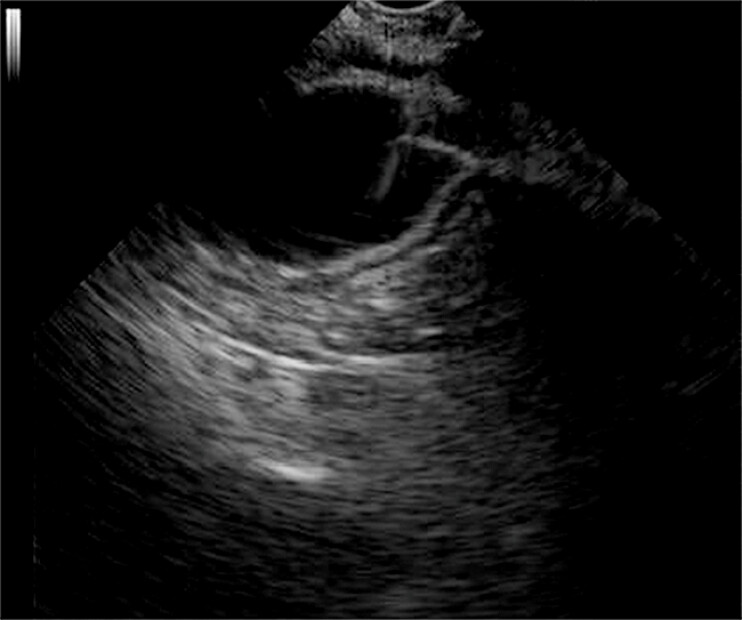
Endoscopic ultrasonogram showed the access of the puncture needle into the intestinal cavity on the oral side of the obstruction.

**Fig. 4:**
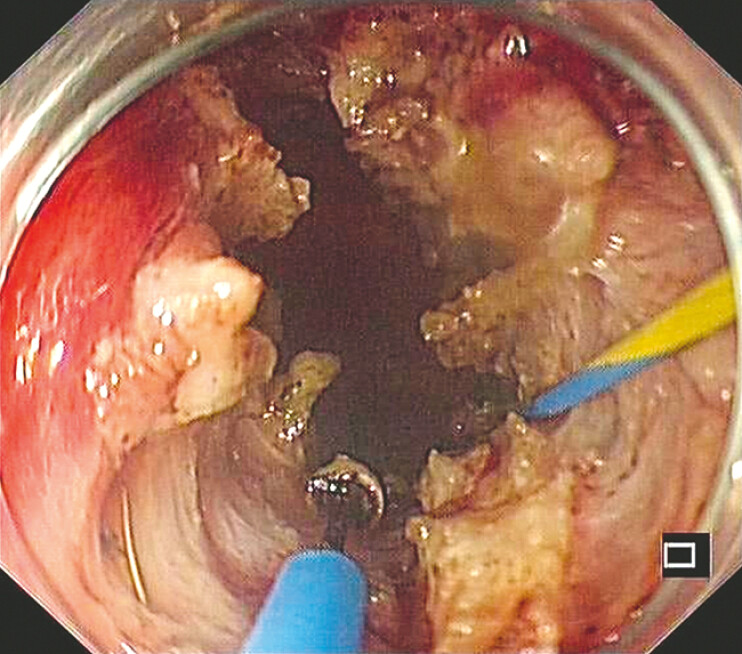
Endoscopic image after radial incision and cutting technique, and dilation to a maximum diameter of 15 mm.

Four more balloon dilation procedures were performed, and no progressive stenosis was revealed ( Fig. 5 ). Eventually, the ileostomy was successfully reversed.

**Fig. 5:**
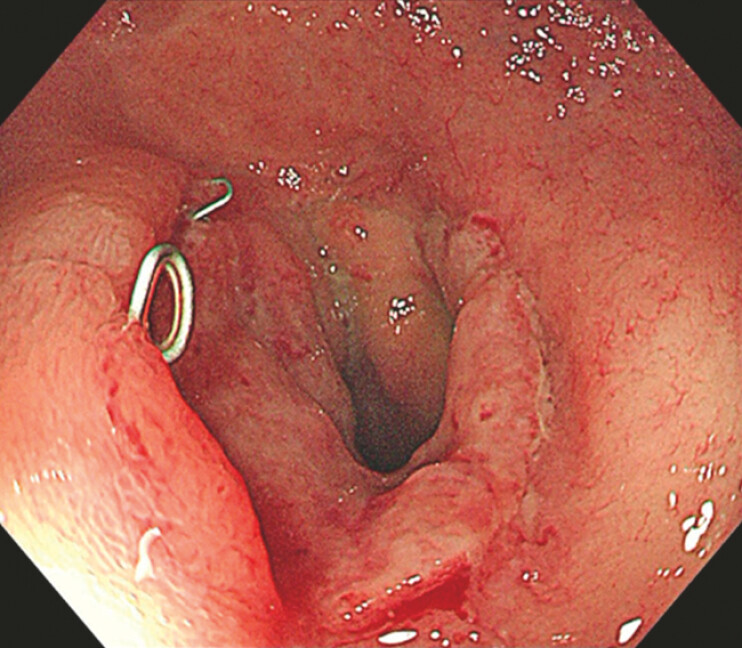
No progressive stenosis was revealed by a follow-up colonoscopy.

This case highlights the utility of EUS-guided recanalization plus ERIC technique, providing a safe, effective, and less invasive option than surgery.

Endoscopy_UCTN_Code_TTT_1AQ_2AF

## Notes

Endoscopy E-Videos https://eref.thieme.de/e-videos
*E-Videos* is an open access online section of the journal *Endoscopy* , reporting on interesting cases and new techniques in gastroenterological endoscopy. All papers include a high-quality video and are published with a Creative Commons CC-BY license. Endoscopy E-Videos qualify for HINARI discounts and waivers and eligibility is automatically checked during the submission process. We grant 100% waivers to articles whose corresponding authors are based in Group A countries and 50% waivers to those who are based in Group B countries as classified by Research4Life (see: https://www.research4life.org/access/eligibility/ ). This section has its own submission website at https://mc.manuscriptcentral.com/e-videos .
